# Prognostic Role of Mucin Antigen MUC4 for Cholangiocarcinoma: A Meta-Analysis

**DOI:** 10.1371/journal.pone.0157878

**Published:** 2016-06-15

**Authors:** Bingmin Li, Haowen Tang, Aiqun Zhang, Jiahong Dong

**Affiliations:** 1 Chinese PLA Medical School, 28 Fuxing Road, Haidian, Beijing, 100853, China; 2 Hospital and Institute of Hepatobiliary Surgery, Chinese PLA General Hospital, Chinese PLA Medical School, 28 Fuxing Road, Haidian, Beijing, 100853, China; The University of Hong Kong, HONG KONG

## Abstract

**Background and Objective:**

Surgery carries the best hope for cure in the treatment of cholangiocarcinoma (CC), whereas surgical outcome is not fully satisfactory. Bio-molecular markers have been used to improve tumor staging and prognosis prediction. Mucin antigen MUC4 (MUC4) has been implicated as a marker for poor survival in various tumors. However, prognostic significance of MUC4 for patients with CC remains undefined. The aim of the present meta-analysis was to investigate the association between MUC4 expression and overall survival (OS) of patients with resected CC.

**Methods:**

The meta-analysis was conducted in adherence to the MOOSE guidelines. PubMed, Embase databases, Cochrane Library and the Chinese SinoMed were systematically searched to identify eligible studies from the initiation of the databases to April, 2016. OSs were pooled by using hazard ratio (HR) with corresponding 95% confidence interval (CI). Random effect models were utilized because of the between-study heterogeneities.

**Results:**

Five studies reporting on 249 patients were analyzed: 94 (37.75%) were in positive or high expression group and 155 (62.25%) in negative or low expression group. The pooled HR for positive or high expression group was found to be 3.04 (95% CI 2.25–4.12) when compared with negative or low expression group with slight between-study heterogeneities (I^2^ 3.10%, P = 0.39). The result indicated that a positive or high expression level of MUC4 was significantly related to poor survival in patients with resected CC. A commensurate result was identified by sensitivity analysis. The main limitations of the present meta-analysis were the rather small size of the studies included and relatively narrow geographical distribution of population.

**Conclusion:**

The result of this meta-analysis indicated that a positive or high expression level of MUC4 was significantly related to poor survival in patients with resected CC.

## Introduction

Cholangiocarcinoma (CC) is a primary tumor arising from the ductal epithelium of the biliary tree. It features markers of cholangiocyte differentiation and carries late diagnoses and poor outcomes [1]. Despite the fact that it is a rare disease accounting for less than 2% of all human malignancies, CC is second (representing about 15% of all liver malignancies) only to hepatocellular carcinoma—the most common type of hepatic malignancy [2,3]. According to anatomical location, contemporary spectrum of CC includes three broad categories (intrahepatic, hilar and extrahepatic). Surgery remains to be the preferred option for all categories and is shown to carry the best hope for cure in the treatment of CC. However, surgical result is not fully satisfactory, with 5-year survival rate ranging from 30% to 41% [4,5]. Traditional clinicopathological factors influencing overall survival (OS) in operable patients have been well delineated, such as TNM stage, resection margin, vascular or neural invasion [6–9]. With the great improvement in tumor biology, bio-molecular markers from biopsy, serum sample, or postoperative specimen may be able to further predict tumor behavior, thus helping inform the patient and clinician in the aspects of either decision-making process or prognosis-predicting efficacy.

Mucins are high molecular weight glycoproteins synthesized by epithelial cells mainly in gastrointestinal, respiratory, genitourinary, and biliary tracts [10]. They are deemed to play important roles in cell protection, repair and survival. During recent years, appropriately 20 human mucins have been identified and classified into two categories: secreted mucins (MUC2, MUC5AC, MUC5B, MUC6, and MUC7) and membrane-anchored mucins (MUC1, MUC3, MUC4, MUC13, MUC15, MUC16, and MUC17) [11,12]. Among them, MUC4, first identified as a tracheobronchial mucin in 1991 [13], has been demonstrated to be with various functional roles in tumor progression and metastasis, and thus may serve as a potential predictor of tumor prognosis. To date, overexpression of MUC4 has been proven to be in relationship with poor survivals in various tumors (lung, esophagus, pancreas and colorectum) [14–17]. However, prognostic significance of MUC4 expression for patients with CC stays undefined [18]. Given this, a meta-analysis was conducted to reveal the influence of MUC4 expression on OS of patients with resected CC.

## Methods

The meta-analysis was conducted in adherence to the recommendations of the Meta-analysis of Observational Studies in Epidemiology group (MOOSE) guidelines [19]. All vital stages of the analysis were carried out separately by two reviewers.

### Study Selection

A systematic literature search of PubMed, Embase databases, Cochrane Library and the Chinese SinoMed was performed to select relevant articles from the initiation of the databases to April, 2016. No additional restrictions were applied to the searches with regard to region, publication type or language. The following medical subject headings (MeSH) or keywords were used: ‘‘Bile Duct Neoplasms,” “Cholangiocarcinoma,” “Cholangiocellular Carcinoma,” “Bile duct,” “Carcinoma,” “Cancer,” “Mucin4,” ‘‘MUC4,” “Prognosis” and “Survival.” In addition, the references given in the retrieved papers were manually checked for further relevant articles. In the case of repeated studies describing the same group of population, only the most recent or highest in quality was included. The latest search was performed on April 10, 2016. To ensure the reliability and verifiability of our analysis, eligible studies were identified in accordance with the following inclusion and exclusion criteria. Inclusion criteria were: (1) postoperative patients with pathologically confirmed diagnosis of CC. (2) MUC4 expression tested by immunohistochemistry (IHC) staining and included as a variable in outcome analysis. (3) stratification of MUC4 expression into positive and negative (or high expression and low expression) groups. (4) survival hazard ratio (HR, describing a summary statistic for censored outcomes) of positive/negative or high expression/low expression available or obtainable from other information presented. A study must meet all 4 inclusion criteria for inclusion. Exclusion criteria were: (1) nonhuman experiments. (2) review articles, letters, case reports, editorials or comments and conference abstracts and studies irrelevant to our topic. (3) focused on a set of mucins rather than MUC4 alone. (4) survival HRs not stated or impossible to calculate. A study meeting any of the 4 exclusion criteria was excluded.

**Fig 1:**
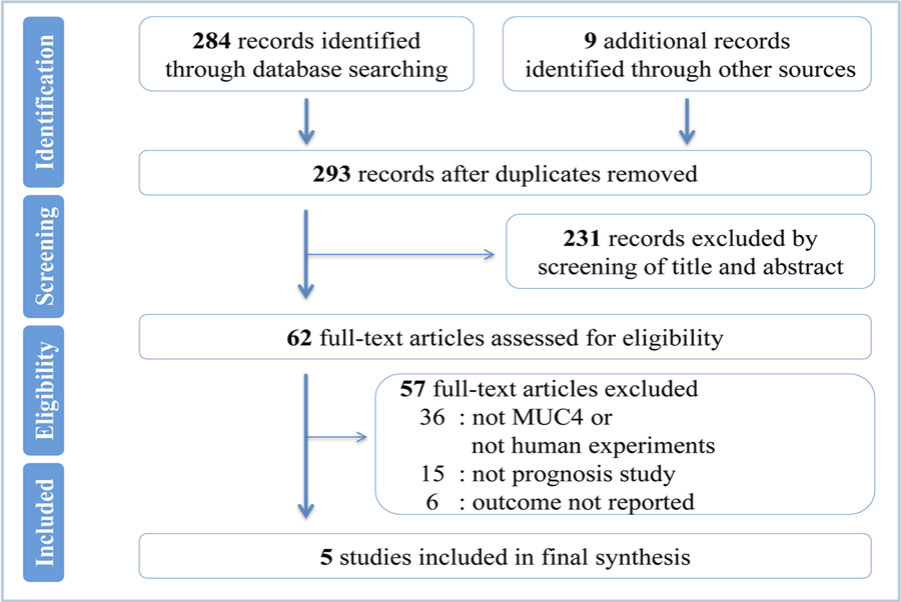
Flowchart of the study selection. Systematic search and selection of relevant articles.

A flowchart of the study selection was shown in Fig 1. The search returned a total of 293 references. By meticulously screening titles and abstracts, 231 references were eliminated. Among the remaining 62 potentially appropriate studies, 57 were excluded by full text analysis for matching one of the exclusion criteria. Finally, 5 studies reporting on 249 patients were eligible to be included in the present meta-analysis [18,20–23].

### Data Extraction and Definition

The following relevant parameters were extracted and summarized independently by two reviewers (BML and HWT) from each study included in the meta-analysis: first author, year of publication, type of study, study region, recruitment period, total patients, mean or median age, proportion of male patients, antibody for MUC4 staining, cut-off value, surveillance endpoint, survival HR, and follow-up length. At the same time, each article included was graded by the Newcastle-Ottawa Scale (NOS) that was mainly concerned with 3 aspects (selection of patients, comparability of groups, and assessment of outcomes). To ensure accuracy and minimize bias, any disagreement was settled through consensus discussion.

### Outcomes Comparison and Statistical Analysis

For comparison of OS, HR with corresponding 95% confidence interval (CI) was used. HR value (reference: negative or low expression group) greater than 1 indicated a strong association between positive or high expression and poor outcome. Random effect models were used because of between-study heterogeneities (explored by I^2^). STATA statistical software (version 12.0, Stata Corporation, College Station, TX, USA) was utilized to conduct the meta-analysis. Begg’s funnel plot and Egger’s tests were used to assess publication bias. Sensitivity analysis was performed by omitting studies included one by one. A P value less than 0.05 was considered statistically significant. If additional key data were absent in the article, the corresponding author of each report was contacted by e-mail.

## Results

### Study Selection and Patients Characteristics

All the 5 studies finally included were retrospective nonrandomized studies published between 2004 and 2012, with a total of 249 patients, of which 94 (37.75%) were in positive or high expression group and 155 (62.25%) in negative or low expression one. The mean or median age of patients included was in 50s or 60s. The proportions of male patients ranged from 50.98% to 67.14%. The sample size for these studies varied from 27 to 70. The studies were conducted in Japan (3 studies) and China (2 studies). The maximum follow-up length of the studies ranged from 60 to 100 months. Study characteristics and quality scoring were summarized in S1 Table.

### Survival Hazard Ratios

**Fig 2:**
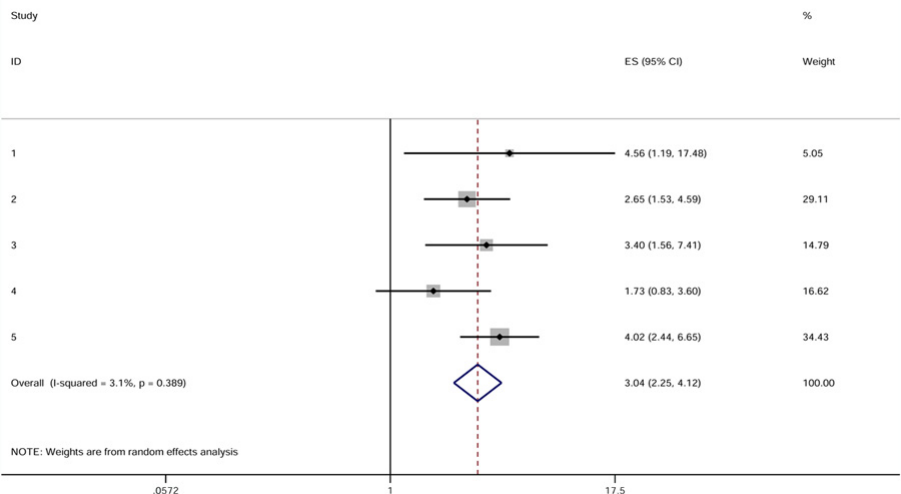
Results of the meta-analysis on pooled HR values. Each square denotes the HR for that trial comparison with the horizontal lines showing the 95% CIs. The size of the square is directly proportional to the amount of information contributed by the trial. The hollow blue diamond gives the pooled HR from the random effect model; the centre of this diamond denotes the HR and the extremities the 95% CI.

The pooled HR for positive or high expression group was found to be 3.04 (95% CI 2.25–4.12) when compared with negative or low expression group with slight between-study heterogeneities (I^2^ 3.10%, P = 0.39). The result indicated that a positive or high expression level of MUC4 was significantly related to poor survival in patients with resected CC. Fig 2 illustrated the results of the meta-analysis.

### Analysis of Sensitivity and Test for Publication Bias

**Fig 3:**
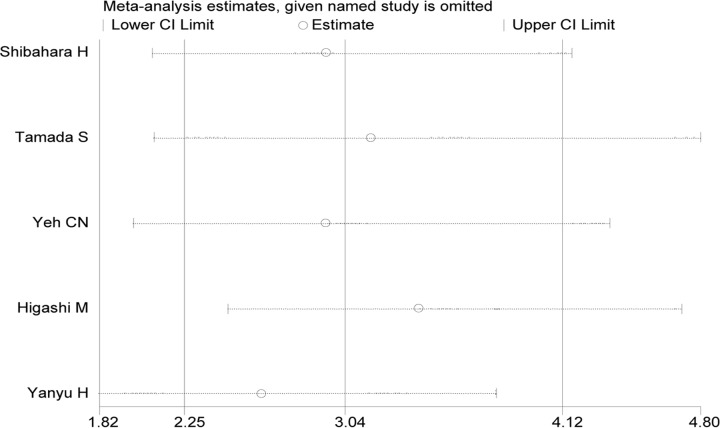
Result of sensitivity analysis. The middle vertical line indicates the combined HR, and the two vertical lines represent the corresponding 95% CI values. The middle small circle and two ends of the dotted lines indicate the pooled HR and 95% CI values, respectively, when the study on the left was omitted after each round of analysis.

**Fig 4:**
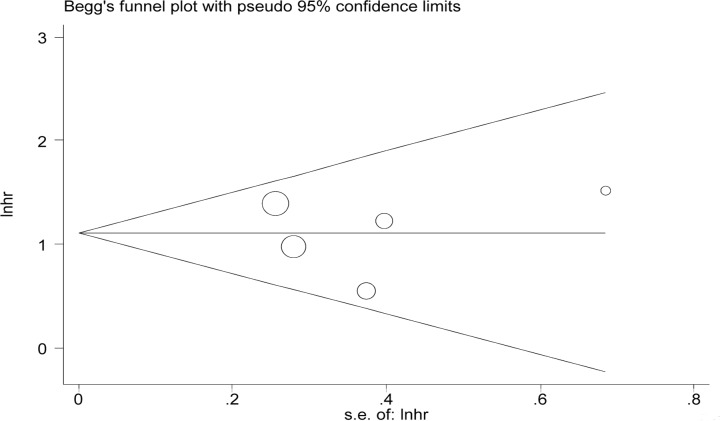
Begg's funnel plot to evaluate OS. Funnel plot showing symmetry indicative of no evidence of publication bias for OS.

By omitting studies included sequentially, a sensitivity analysis aiming to evaluate the impact of a single study on the overall pooled HR was performed. No significant changes of HR values were produced by exclusion of any single study, with a range from 2.62 to 3.40 (Fig 3). There was no evident publication bias by Eegg’s test (P = 0.95), with symmetry in Begg’s funnel plot as shown in Fig 4. However, this should be interpreted with much caution for the small number of included studies.

## Discussion

MUC4 is found aberrantly expressed in various malignancies and has been demonstrated to be a potential prognostic marker for some tumors [14–17,24,25]. However, studies discussing the prognostic role of MUC4 expression for patients with CC were limited. This meta-analysis has reviewed the relationship between MUC4 expression and OS of patients with resected CC. The results showed that patients with positive or high expression of MUC4 carried a survival inferiority when compared to those with negative or low expression levels (HR 3.04, 95% CI 2.25–4.12), and indicated that MUC4 might be a potential bio-molecular marker to predict prognosis of patients with resected CC.

The above results might be mainly caused by the particular features and related molecular mechanisms of MUC4, which promoted tumor growth and progression. MUC4 could be found normally expressed in normal tissues of the respiratory tract, stomach, small intestine, colon, and endocervix [26–29]. And it has been recently proved to be a novel intramembrane ligand for receptor tyrosine kinase ErbB2 [30,31]. Through forming a MUC4 complex with ErbB2 receptor, the transmembrane subunit of MUC4 showed specific binding to ErbB2 and hence induced the tyrosine phosphorylation of ErbB2 [32,33]. Specifically, MUC4 is the only ligand that has been characterized for ErbB2 [34–36]. Furthermore, the disorganization resulting from MUC4 overexpression might pose a decreased effect on adhesion to other cells as well as the extracellular matrix, and therefore promoted the migration and metastasis of tumor cell [37]. In addition, as reported, the presence of MUC4 on tumor cell would cloak the surface epitopes to the cytotoxic immune cells, which helped the tumor cells escape from immunological attack [38,39]. Taken together, the features and mechanisms aforementioned explained the clinical finding that survival inferiority was found in patients with positive or high expression of MUC4. This kept consistent with the findings from Matull WR who found that increased expression of MUC4 was highly specific to biliary tract cancers and significantly determined a poorer long-term outcome [40]. In his study, aberrant expression of MUC4 was found in 37% of the biliary tract cancer specimens and 29% of the primary sclerosing cholangitis bile samples, whereas such expression could be hardly seen in benign biliary tissues. Furthermore, MUC4 expression predicted a shorter survival length in patients with biliary tract cancers. Median survival length of positive expression group was only 5.2 months when compared to 11.8 months for negative expression group [40]. In keeping with the above results, our study again revealed the impact of MUC4 expression on long-term outcome in patients with CC.

Our present study has 3 main strengths. (1) By relatively strict patient-selecting criteria (inclusion and exclusion), a total of 249 patients were included, forming a substantial retrospective cohort from which to make clinical reasonable assumptions about patients. (2) As to time to event data, the best option of using HR value to perform the pooled analysis of OS effect was conducted. (3) On sensitivity analysis, a similar result was produced and thus confirmed the overall finding.

In spite of the above-mentioned improvements, some limitations of the present study should be taken into consideration. The main limitation was that the size of the studies included was rather small for the relative rarity of CC. Besides, all the data were obtained from the Asian hospitals or medical centers. Potentially, the existence of biological distinction in tumor behavior between Asian and non-Asian populations might reduce the reliability and applicability of the results. In addition, the lack of relevant data did not permit subgroup analysis (intrahepatic, hilar and extrahepatic types evaluated separately) to be conducted. Finally, although MUC4 expressions were all evaluated by IHC, differences in staining protocol, antibody usage and cutoff value definition would probably lead to considerable heterogeneity and variability. Nevertheless, the current study undoubtedly represents one more step in summarizing the released evidence concerning this topic.

## Conclusion

In summary, the result of this meta-analysis suggested that an elevated expression of MUC4 in patients with resected CC was closely correlated with poor long-term survival. Hence, MUC4 can be used as a potential prognostic marker for patients who had surgical resection of CC. Further multicenter prospective studies are required to back up the conclusion.

## Supporting Information

S1 PRISMA Checklist PRISMA checklist. (DOC)

S1 Table Characteristics of included studies. NOS score: Newcastle-Ottawa Scale score; R: retrospective; IHC: Immunohistochemistry; 8G7: 8G7 clone antibody; 1G8: 1G8 clone antibody; PcAb: polyclonal antibody. ^a^ Cut off refers to the percentage of cells with positively staining nuclei unless stated otherwise. ¶: value is mean with range in parenthesis; †: value is median with range in parenthesis.
(DOC)
