# Expression of Concern: Prognostic Role of Mucin Antigen MUC4 for Cholangiocarcinoma: A Meta-Analysis

**DOI:** 10.1371/journal.pone.0285348

**Published:** 2023-04-28

**Authors:** The PLOS ONE Editors

After this article was published, similarities were noted between this article [1] and submissions by other research groups which call into question the validity and provenance of the reported results.

In response to queries about these concerns, the first authors provided the underlying data in S1 File. During editorial follow-up, the following errors were noted:

- The upper limit of 95% CI (6.265) was input as 3.384 in error for reference 20 [2]. The first authors stated that the revised data (HR = 2.655 (95% CI, 1.125–6.265)) inputted to the STATA software (Version 12) gave rise to a pooled analysis which showed consistent results. An updated version of Fig 3, and an updated Begg’s plot (Fig 4) using the revised data (HR = 2.655 (95% CI, 1.125–6.265)) for reference 20 [2] are provided here. Updated text is also provided below:
  ○ Abstract: The pooled HR for positive or high expression group was found to be 3.14 (95% CI 2.26–4.35) when compared with negative or low expression group with slight between-study heterogeneities (I^2^ 3.96%, P = 0.41).
  ○ Results: Survival Hazard Ratios: The pooled HR for positive or high expression group was found to be 3.14 (95% CI 2.26–4.35) when compared with negative or low expression group with slight between-study heterogeneities (I^2^ 3.96%, P = 0.41).
  ○ Results: Analysis of Sensitivity and Test for Publication Bias: No significant changes of HR values were produced by exclusion of any single study, with a range from 2.62 to 3.40 (Fig 3). There was no evident publication bias by Begg’s test (P = 0.95), with symmetry in Begg’s funnel plot as shown in Fig 4.
  ○ Discussion: The results showed that patients with positive or high expression of MUC4 carried a survival inferiority when compared to those with negative or low expression levels (HR 3.14, 95% CI 2.26–4.35), and indicated that MUC4 might be a potential bio-molecular marker to predict prognosis of patients with resected CC.
- Study IDs were not included in Fig 2. An updated version of Fig 2 with study details added and revised data for reference 20 [2] is provided here.
- The author name in reference 23 [4] was misspelt. The correct citation details are listed below as reference [4].

**Fig 2:**
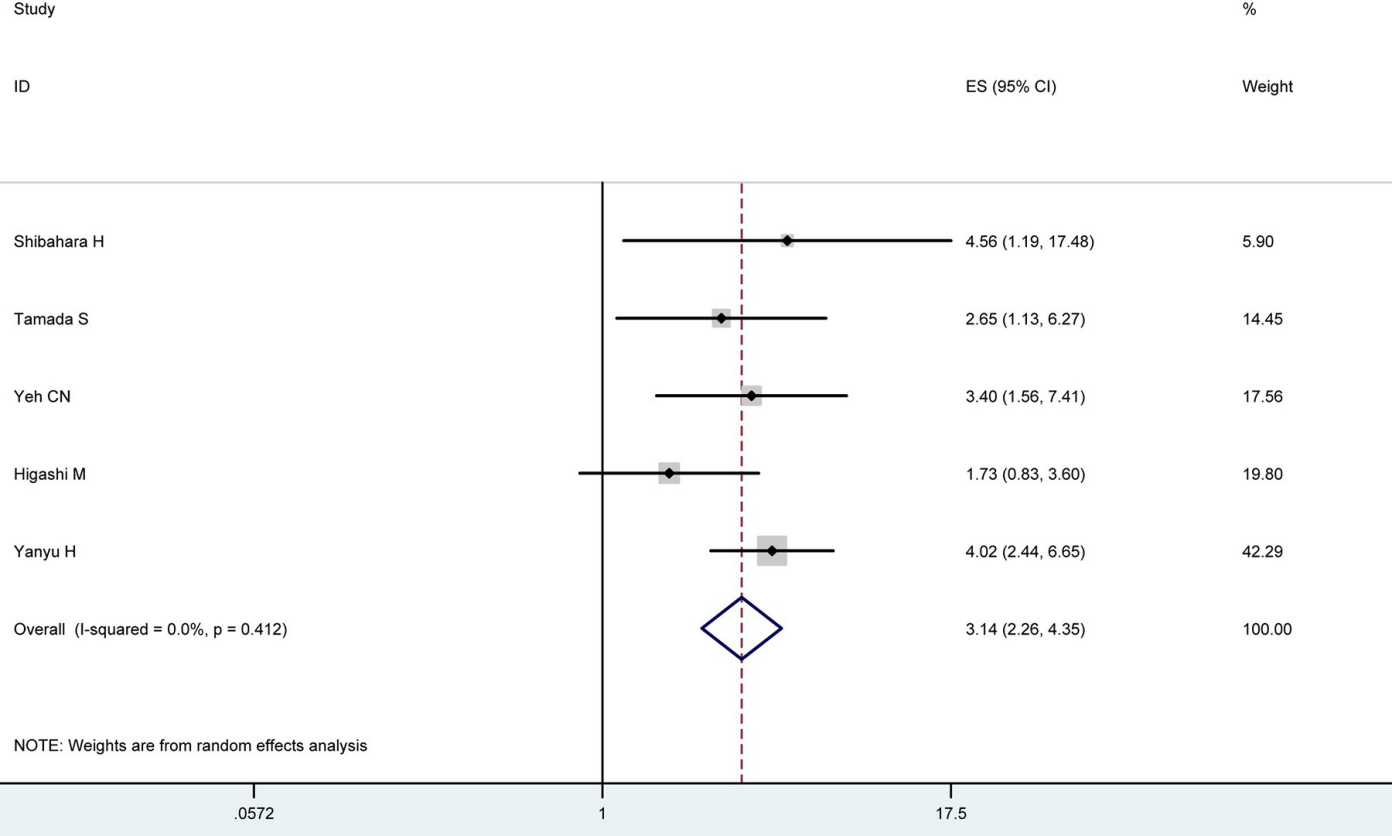
Results of the meta-analysis on pooled HR values. Each square denotes the HR for that trial comparison with the horizontal lines showing the 95% CIs. The size of the square is directly proportional to the amount of information contributed by the trial. The hollow blue diamond gives the pooled HR from the random effect model; the centre of this diamond denotes the HR and the extremities the 95% CI.

**Fig 3:**
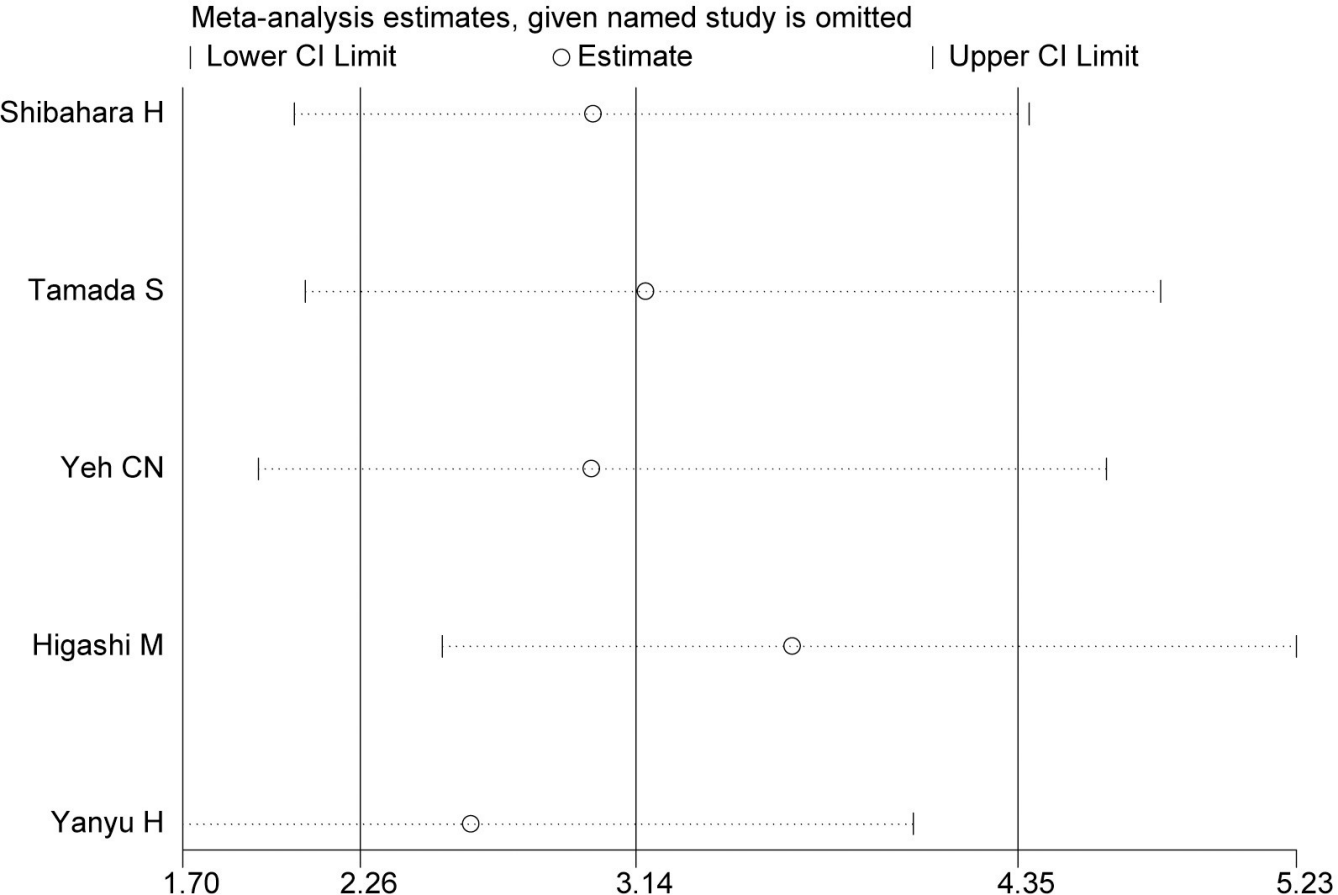
Result of sensitivity analysis. The middle vertical line indicates the combined HR, and the two vertical lines represent the corresponding 95% CI values. The middle small circle and two ends of the dotted lines indicate the pooled HR and 95% CI values, respectively, when the study on the left was omitted after each round of analysis.

**Fig 4:**
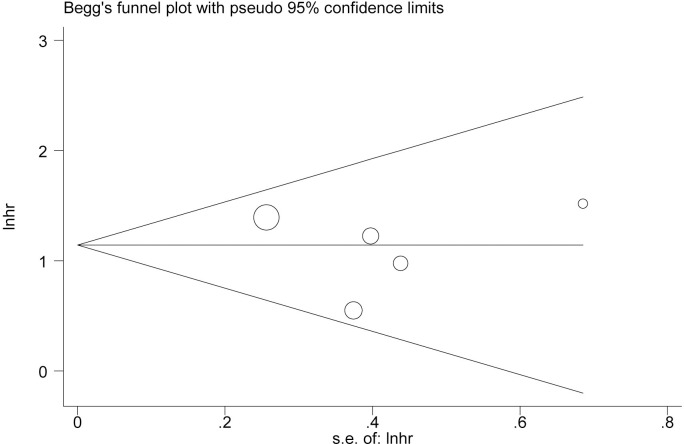
Begg’s funnel plot to evaluate OS. Funnel plot showing symmetry indicative of no evidence of publication bias for OS.

The authors commented on aspects of how data were collected and analyzed for this study, but overall their responses did not fully resolve the concerns.

The *PLOS ONE* Editors issue this Expression of Concern to notify readers of the unresolved concerns discussed above, and to provide the data received from the authors.

## Supporting information

S1 File The underlying data used for the analysis in this article [1]. (7Z)
