# Supplementary material for: Prognostic Role of Mucin Antigen MUC4 for Cholangiocarcinoma: A Meta-Analysis
Source: PLoS One. 2016 Jun 15;11(6):e0157878. doi: 10.1371/journal.pone.0157878 (PMC4909222; doi:10.1371/journal.pone.0157878)
Supplement: S1 Table — NOS score: Newcastle-Ottawa Scale score; R: retrospective; IHC: Immunohistochemistry; 8G7: 8G7 clone antibody; 1G8: 1G8 clone antibody; PcAb: polyclonal antibody. a Cut off refers to the percentage of cells with positively staining nuclei unless stated otherwise. ¶: value is mean with range in parenthesis; †: value is median with range in parenthesis. (DOC) [file pone.0157878.s002.doc]

**Supporting Information**

**S1 PRISMA Checklist.** PRISMA checklist.

| **First author** | **Year** | **Type** | **Region** | **Period** | **Patients characteristics** | | | | **Detecting**  **method** | **Cut**  **Off** ^a^ | **End point** | **HR** | | | **Follow-up**  **(months)** | **NOS**  **score** |
| --- | --- | --- | --- | --- | --- | --- | --- | --- | --- | --- | --- | --- | --- | --- | --- | --- |
|  |  |  |  |  | **Total** | Age | Male (%) | MUC4+ (n) |  |  |  | Source | Value | 95% CI |  |  |
| Shibahara H^18^ | 2004 | R | Japan | 1986-1999 | 27 | 65.3 (45-79)¶ | 59.26% | 10 | IHC-8G7 | 5% | OS | Reported | 4.56 | 1.19-17.48 | 60 | 7 |
| Tamada S^20^ | 2006 | R | Japan | 1990-2000 | 70 | 69.2 (41-88)¶ | 67.14% | 19 | IHC-8G7 | 5% | OS | Reported | 2.66 | 1.13-3.38 | 100 | 8 |
| Yeh CN^21^ | 2009 | R | China | 2000-2004 | 51 | 60 (29-89)† | 50.98% | 13 | IHC-1G8 | 1% | OS | Reported | 3.40 | 1.56-7.41 | 70.1 | 8 |
| Higashi M^23^ | 2012 | R | Japan | 1986-2006 | 63 | 67.4 (41-85)¶ | 52.38% | 19 | IHC-8G7 | 5% | OS | Reported | 1.73 | 0.83-3.60 | - | 7 |
| Yanyu H^22^ | 2012 | R | China | 2006-2010 | 38 | 58 (38-72)† | 57.89% | 33 | IHC-PcAb | 5% | OS | Reported | 4.02 | 2.00-5.46 | 60 | 7 |

**S1 Table Characteristics of included studies**

NOS score: Newcastle-Ottawa Scale score; R: retrospective; IHC: Immunohistochemistry ; 8G7: 8G7 clone antibody; 1G8: 1G8 clone antibody; PcAb: polyclonal antibody. ^a^ Cut off refers to the percentage of cells with positively staining nuclei unless stated otherwise. ¶: value is mean with range in parenthesis; †: value is median with range in parenthesis.
